# Perceptions of buprenorphine barriers and efficacy among nurse practitioners and physician assistants

**DOI:** 10.1186/s13722-022-00321-6

**Published:** 2022-08-09

**Authors:** Barbara Andraka-Christou, Cory Page, Victoria Schoebel, Jessica Buche, Rebecca L. Haffajee

**Affiliations:** 1 School of Global Health Management and Informatics, University of Central Florida, 500 W Livingston Ave, Suite 400, FL Orlando, USA; 2 Department of Internal Medicine, University of Central Florida, 6850 Lake Nona Blvd, Orlando, FL 32827 USA; 3 Behavioral and Physical Health and Aging Services Administration, Department of Health and Human Services, Lansing, MI USA; 4 Present Address: Assistant Secretary for Planning and Evaluation, U.S. Department of Health and Human Services, Washington D.C., USA; 5 Department of Health Management and Policy, School of Public Health, University of Michigan, Ann Arbor, MI USA; 6 School of Public Health, University of Minnesota, Minneapolis, MI USA

**Keywords:** Nurse practitioners, Physician assistants, Buprenorphine, Methadone, Naltrexone, Survey, Barriers, Efficacy, Perceptions, Counseling, Peer support, Detoxification, Prior authorization

## Abstract

**Background:**

Medications for opioid use disorder (MOUDs), including methadone, buprenorphine, and naltrexone, decrease mortality and morbidity for people with opioid use disorder (OUD). Buprenorphine and methadone have the strongest evidence base among MOUDs. Unlike methadone, buprenorphine may be prescribed in office-based settings in the U.S., including by nurse practitioners (NPs) and physician assistants (PAs) who have a federal waiver and adhere to federal patient limits. Buprenorphine is underutilized nationally, particularly in rural areas, and NPs/PAs could help address this gap. Therefore, we sought to identify perceptions of buprenorphine efficacy and perceptions of prescribing barriers among NPs/PAs. We also sought to compare perceived buprenorphine efficacy and perceived prescribing barriers between waivered and non-waivered NPs/PAs, as well as to compare perceived buprenorphine efficacy to perceived naltrexone and methadone efficacy.

**Methods:**

We disseminated an online survey to a random national sample of NPs/PAs. We used Mann–Whitney *U* tests to compare between waivered and non-waivered respondents. We used non-parametric Friedman tests and post-hoc Wilcoxon signed-rank tests to compare perceptions of medication types.

**Results:**

240 respondents participated (6.5% response rate). Most respondents agreed buprenorphine is efficacious and believed counseling and peer support should complement buprenorphine. Buprenorphine was generally perceived as more efficacious than both naltrexone and methadone. Perceived buprenorphine efficacy and prescribing barriers differed by waiver status. Non-waivered practitioners were more likely than waivered practitioners to have concerns about buprenorphine affecting patient mix. Among waivered NPs/PAs, key buprenorphine prescribing barriers were insurance prior authorization and detoxification access.

**Conclusions:**

Our results suggest that different policies should target perceived barriers affecting waivered versus non-waivered NPs/PAs. Concerns about patient mix suggest stigmatization of patients with OUD. NP/PA education is needed about comparative medication efficaciousness, particularly regarding methadone. Even though many buprenorphine treatment patients benefits from counseling and/or peer support groups, NPs/PAs should be informed that such psychosocial treatment methods are not necessary for all buprenorphine patients.

## Introduction

Treatment with medications for opioid use disorder (MOUDs) is effective for reducing mortality and morbidity [1–4]. MOUD options include buprenorphine treatment (BUP), methadone treatment (MET), and naltrexone treatment (NTX). Among MOUDs, methadone and buprenorphine have the strongest evidence base [1] and are generally considered first-line opioid use disorder (OUD) treatments [5]. In the U.S., MET is only available in opioid treatment programs. In contrast, BUP and NTX are available in office-based settings; however, a BUP provider must first obtain a waiver from the federal government (hereinafter “DATA waiver”) and adhere to patient limitations [6]. In 2020, 29% of all U.S. counties lacked any practitioner with a DATA waiver and 51% of small or remote rural areas lacked any practitioner with a DATA waiver [7]. Even when practitioners have a waiver, the vast majority do not prescribe up to their maximum capacity as allowed by law [8, 9], suggesting BUP accessibility remains low.

In 2016, the Comprehensive Addiction and Recovery Act (CARA) [10] permitted physician assistants (PAs) and nurse practitioners (NPs) to become eligible for DATA waivers, which were previously reserved for physicians. NPs and PAs have significantly contributed to recent expansions in buprenorphine supply [11–13]. Almost 8% of small or remote rural counties only have an NP or PA with a waiver [7]. Therefore, DATA-waivered NPs and PAs represent an opportunity to increase buprenorphine accessibility [11], particularly in rural areas.

Little is known, however, about NP/PA perceptions of BUP efficacy and barriers [14], with most studies of BUP barriers having been focused on physicians [15]. A previous study examined BUP barriers among NPs/PAs [14], but did not compare waivered to non-waivered practitioners, who may face different types of barriers that require different policy solutions. For example, practitioners who are already waivered may be more likely to perceive insurance policies as a barrier as compared to lack of training, with the latter potentially addressed through educational requirements for the DATA waiver. Information about NP/PA prescribing barriers is particularly important in light of new federal guidelines which still require a waiver but permit NPs and PAs to obtain the waiver without specialized education when prescribing to fewer than 30 patients [16].

Furthermore, little is known about how NP/PA perceptions of BUP efficacy differ from those for MET and NTX. Perceptions of efficacy for all three MOUDs are important, because office-based NPs/PAs can prescribe BUP and NTX, and can refer patients to MET [10]. The appropriate treatment may differ from patient to patient, and consistent with person-centered care principles, patients with OUD should receive accurate information about all three options from NPs/PAs [17].

Therefore, our study had two aims: First, to examine NP/PA perceptions of BUP efficacy and barriers, comparing among waivered and non-waivered practitioners; second to compare NP/PA perceptions of efficacy and barriers across all three MOUD options (i.e., MET, BUP, NTX.)

## Methods

### Sample

We received ethical approval from the University of Michigan Institutional Review Board for this research. Based on a review of the literature, we created and administered an online, Qualtrics™ survey. The original sample frame included all NPs and PAs nationwide whom the medical marketing companies RediData and ExactData had contact information. We then distinguished these providers as “high-frequency” and “low-frequency” MOUD prescribers: high-frequency providers had addiction treatment specializations and/or worked in SUD clinics, whereas low-frequency providers had no such specialty and worked in general or family practice settings. The final, random sample of 3,711 NPs/PAs received survey invitations by email in summer 2018. Two reminder emails were sent to encourage participation. Respondents received a $25 incentive upon survey completion.

### Measures

We received 264 responses. We removed 24 responses from participants who completed less than half the survey or were not a NP/PA. The six-part survey covered the following topics: respondent demographics (gender, race/ethnicity, and education), professional characteristics and practice settings, screening practices for SUD, SUD maintenance practices, MOUD knowledge and usage, and experienced treatment barriers. Due to sample size, Likert answer sets were recoded from five response categories (“Strongly Disagree”, “Disagree”, “Neither Disagree nor Agree”, “Agree”, and “Strongly Agree) to three response categories (“Disagree,” “Neither,” and “Agree”).

### Analyses

For demographic and professional characteristic data, we used chi-square tests for categorical variables and t-tests for continuous variables to determine the differences between NPs and PAs, as well as the difference between waivered and non-waivered respondents.

Given the data’s non-parametric, ordinal nature, Mann–Whitney U tests were used to assess BUP efficacy and barrier responses between waivered and non-waivered providers. We also performed non-parametric Friedman tests to detect differences in the providers’ perceived efficacy of BUP, MET, and NTX. Post-hoc Wilcoxon signed-rank tests with Holm-Bonferroni corrections were used to further determine which MOUDs differed from one another. The Holm-Bonferroni method was used to adjust for the 27 Wilcoxon signed-rank tests to account for the multiple comparisons that can increase the family-wise error rate. All p-values were two-tailed with an alpha of 0.05. STATA 15 software was used for all statistical analyses.

## Results

### Respondent characteristics

The final sample had 240 respondents (6% response rate), with a nearly even distribution of 118 NPs (51%) and 122 PAs (49%). Table 1 presents the demographic information and professional characteristics of the practitioners included in the final sample. The most frequent demographic characteristics were as follows: female (77%); white (84%); and had completed a master’s degree (78%). Respondents also had the following professional characteristics: have practiced for 26 or more years (32%); practiced in a family medicine setting (31%); received training in dual diagnosis disorders (44%); and saw an average of 331 patients per month.

**Table 1:** Demographic information and professional characteristics of nurse practitioners and physician assistants with and without a DATA-waiver

Participant characteristics^a^	Total	Waivered	Not waivered	*P*^b^
Total, n (%)	240 (100)	108 (46)	129 (54)	
Sex, n (%)				0.48
Female	177 (77)	78 (45)	97 (55)	
Male	54 (23)	27 (50)	27 (50)	
Race/ethnicity, n (%)				0.13
White	194 (84)	89 (46)	103 (54)	
Black/African American	11 (5)	8 (73)	3 (27)	
Other/Multi-racial	25 (11)	9 (36)	16 (64)	
Highest level of education, n (%)				**0.01**
Doctorate	22 (9)	16 (73)	6 (27)	
Master’s degree	181 (78)	82 (46)	97 (54)	
Other	29 (13)	8 (28)	21 (72)	
Provider				** < 0.001**
Nurse practitioner	122 (51)	78 (67)	38 (33)	
Physician assistant	118 (49)	30 (25)	91 (75)	
Years practicing, n (%)				0.76
0–5	47 (20)	22 (47)	25 (53)	
6–10	41 (18)	37 (49)	38 (51)	
11–15	30 (13)	17 (42)	23 (58)	
16–20	19 (8)	11 (38)	18 (62)	
21–25	21 (9)	7 (37)	12 (63)	
26 +	75 (32)	11 (55)	9 (45)	
Practice facility, n (%)				** < 0.001**
Family medicine (outpatient)	75 (31)	20 (27)	55 (73)	
Pain medicine practice (outpatient)	24 (10)	14 (61)	9 (39)	
Substance use disorder treatment programs	21 (9)	20 (95)	1 (5)	
General hospital or emergency department	16 (7)	2 (12)	14 (88)	
Other	39 (16)	8 (22)	29 (78)	
Multiple practice sites	65 (27)	44 (68)	21 (32)	
Number of patients seen per month, mean (SD)	330.80 (689.35)	367.24 (707.33)	299.40 (682.57)	** < 0.001**
Specialization, n (%)				** < 0.001**
Dual diagnosis disorders (addiction/mental illness)	40 (17)	33 (85)	6 (15)	
Family medicine	28 (12)	11 (39)	17 (61)	
Substance use disorders/addiction	27 (11)	24 (89)	3 (11)	
Mental illness disorders	14 (6)	6 (43)	8 (57)	
Other	70 (29)	14 (21)	54 (79)	
Multiple specializations	24 (10)	14 (58)	10 (42)	
No specialization	37 (15)	6 (16)	31 (84)	
Received training in past 3 years, n (%)				** < 0.001**
Dual diagnosis disorders (addiction/mental illness)	105 (44)	61 (58)	44 (42)	
Substance use disorders/addiction	61 (26)	41 (69)	18 (31)	
Mental illness disorders	10 (4)	1 (10)	9 (90)	
No training in any of the above	61 (26)	5 (8)	55 (92)	

Bold values indicate significance at an alpha of 0.05

^a^Totals vary due to missing values

^b^P-value from chi-square tests for categorical variables and two-sample t-tests for continuous variables

Fifty-four percent of providers did not have a DATA waiver. Non-waivered providers, as compared to waivered, were significantly more likely to be a physician assistant (*P* < 0.001) and work in a family medicine setting (*P* < 0.001), were significantly less likely to have a behavioral health specialization (*P* < 0.001) and have received behavioral health training (*P* < 0.001), and saw fewer patients per month (*P* < 0.001).

### Perceptions of BUP effectiveness: Waivered versus non-waivered practitioners

Table 2 displays the percentage of waivered and non-waivered respondents who agreed with each efficacy statement. Nearly all (> 90%) waivered respondents agreed BUP decreases risk of death from overdose, decreases cravings, and prevents relapse; most (71–80%) non-waivered respondents also agreed with these statements. Almost all DATA-waivered providers (90%) believed BUP works well for patients with co-occurring mental health disorders, while only 64% of non-waivered providers believed similarly. Fewer than half of waivered and non-waivered NPs/PAs believed BUP is appropriate for patients with unstable OUD conditions (48% and 27%, respectively.) Among both waivered and non-waivered populations, the majority (> 86%) agreed BUP should be supplemented with counseling, should be supplemented with peer support, and that BUP efficacy is improved with counseling. Waivered practitioners were significantly more likely than non-waivered practitioners to agree BUP decreases risk of death from overdose (*U* = 5095, *P* < 0.001), decreases cravings (*U* = 4528, *P* = 0.01), decreases relapse (*U* = 4534, *P* < 0.001), works well for individuals with co-occurring mental health disorders (*U* = 4886, *P* < 0.001), and is appropriate for patients with unstable OUD conditions (*U* = 4398, *P* = 0.004).

**Table 2:** Variations in the perceived efficacy of providing buprenorphine between DATA-waivered providers and non-waivered providers

Perceived efficacy^a^	Waivered				Non-waivered				Mann–Whitney		
	Disagree n (%)	Neither n (%)	Agree n (%)	Mdn	Disagree n (%)	Neither n (%)	Agree n (%)	Mdn	r	U	*P*^b^
Decreases risk of death from an opioid overdose	1 (1)	3 (3)	101 (96)	3	6 (8)	16 (20)	56 (72)	3	0.34	5095	** < 0.001**
Decreases cravings for opioids	1 (1)	4 (4)	101 (95)	3	3 (4)	10 (13)	63 (83)	3	0.20	4528	**0.01**
Decreases rates of relapse	2 (2)	9 (8)	95 (90)	3	4 (5)	17 (24)	51 (71)	3	0.24	4534	** < 0.001**
Works well in clients with co-occurring mental health disorders	2 (2)	9 (8)	96 (90)	3	3 (4)	23 (32)	47 (64)	3	0.30	4886	** < 0.001**
Should be supplemented by mental health counseling	4 (4)	7 (7)	88 (89)	3	1 (1)	6 (8)	67 (91)	3	0.03	3594	0.69
Should be supplemented by participation in peer support groups	3 (3)	10 (9)	93 (88)	3	2 (3)	8 (11)	63 (86)	3	0.02	3923	0.79
Efficacy is improved by adding mental health counseling	2 (2)	8 (7)	96 (91)	3	0	6 (8)	70 (92)	3	0.03	3960	0.69
Appropriate for unstable patients	22 (21)	32 (31)	50 (48)	2	24 (35)	26 (38)	18 (27)	2	0.22	4398	**0.004**

*Mdn* median

^a^Totals vary due to missing values

^b^Bold values indicate significance at an alpha of 0.05

### Perceptions of BUP barriers: Waivered versus non-waivered practitioners

Table 3 displays the percentage of waivered and non-waivered respondents who agreed with each barrier statement. The most common barriers indicated by waivered practitioners were prior authorization requirements (29%), insufficient detoxification access (30%), and insufficient psychosocial support (23%). The most common barriers indicated by non-waivered practitioners were insufficient expertise (40%), insufficient detoxification access (38%), and insufficient psychosocial support (37%). Non-waivered practitioners were significantly more likely to agree with each of the following barriers: BUP would unfavorably affect patient mix (*U* = 2250, *P* = 0.02), insufficient training (*U* = 1922, *P* = 0.002), insufficient time (*U* = 1984, *P* = 0.002), insufficient experience (U = 1922, *P* < 0.001), and insufficient staff support (U = 2073, *P* = 0.004).

**Table 3:** Variations in the perceived barriers for providing buprenorphine between DATA-waivered providers and non-waivered providers

Perceived barriers^a^	Waivered				Non-waivered				Mann–Whitney		
	Disagree n (%)	Neither n (%)	Agree n (%)	Mdn	Disagree n (%)	Neither n (%)	Agree n (%)	Mdn	r	U	*P*^b^
Concerns about diversion	20 (24)	49 (60)	13 (16)	2	16 (22)	43 (59)	14 (19)	2	0.05	2856	0.58
Lack of patient interest	36 (44)	40 (49)	6 (7)	2	30 (40)	39 (53)	5 (7)	2	0.03	2949	0.74
Law enforcement oversight	46 (57)	31 (39)	3 (4)	1	33 (48)	27 (40)	8 (12)	2	0.12	2393	0.16
Professional licensing board oversight	48 (57)	28 (33)	8 (10)	1	28 (42)	31 (46)	8 (12)	2	0.14	2394	0.08
Treatment patients would unfavorably affect my patient mix	57 (73)	18 (23)	3 (4)	1	39 (56)	26 (37)	5 (7)	1	0.19	2250	**0.02**
Co-workers do not support provision of buprenorphine treatment in my practice	58 (73)	13 (16)	9 (11)	1	35 (55)	20 (32)	8 (13)	1	0.16	2131	0.06
Managers/administrators do not support provision of buprenorphine treatment in my practice	60 (73)	16 (19)	7 (8)	1	38 (61)	13 (21)	11 (18)	1	0.13	2248	0.12
Reimbursement rates	38 (48)	29 (36)	13 (16)	2	28 (48)	25 (43)	5 (9)	2	0.04	2428	0.61
Insurance prior authorization requirements	18 (23)	38 (48)	23 (29)	2	12 (20)	37 (63)	10 (17)	2	0.07	2509	0.40
Insufficient training	39 (49)	35 (44)	6 (7)	2	20 (30)	28 (43)	18 (27)	2	0.25	1922	**0.002**
Insufficient time	34 (42)	37 (46)	10 (12)	2	14 (21)	35 (52)	18 (27)	2	0.25	1984	**0.002**
Insufficient staff support	35 (42)	37 (45)	11 (13)	2	16 (24)	31 (46)	20 (30)	2	0.24	2073	**0.004**
Insufficient experience	40 (48)	33 (40)	10 (12)	2	15 (21)	27 (39)	28 (40)	2	0.35	1801	** < 0.001**
Insufficient resources for patient psychosocial support within the community or my practice	18 (21)	47 (56)	19 (23)	2	12 (16)	36 (47)	28 (37)	2	0.15	2696	0.06
Insufficient resources for patient detoxification within the community or my practice	17 (21)	40 (49)	25 (30)	2	11 (14)	37 (48)	29 (38)	2	0.10	2836	0.23

*Mdn* median

^a^Totals vary due to missing values

^b^Bold values indicate significance at an alpha of 0.05

### Comparison of perceived efficacy by medication

The results of the Friedman test indicate a significant difference between the perceived efficacy of BUP, MET, and NTX in decreasing the risk of death from an opioid overdose (39.58(2), *P* < 0.001), decreasing opioid cravings (36.03(2), *P* < 0.001), decreasing rates of relapse (25.04(2), *P* < 0.001), and working well for individuals with co-occurring mental health disorders (32.29(2), *P* < 0.001; Table 4). Additionally, there was a significant difference between BUP, MET, and NTX regarding providers’ perceptions that these medications should be supplemented with counseling (7.79(2), *P* = 0.02), supplemented with peer support groups (7.80(2), *P* = 0.02), are more effective if supplemented with counseling (14.90(2), *P* = 0.001), are appropriate for unstable patients (19.37(2), *P* = 0.001), and are often diverted or misused (91.95(2), *P* < 0.001).

**Table 4:** Differences in nurse practitioner and physician assistant perceived efficacy of buprenorphine, methadone, and naltrexone

Perceived efficacy	Formulation			df	Friedman test statistic Q	P^a^
	Buprenorphine n (%)	Methadone n (%)	Naltrexone n (%)			
Decreases risk of death from an opioid overdose						
Agree	157 (86)	119 (58)	117 (71)	2	39.58	** < 0.001**
Neither	19 (10)	44 (22)	42 (25)			
Disagree	7 (4)	41 (20)	6 (4)			
Decreases cravings for opioids						
Agree	164 (90)	149 (73)	112 (67)	2	36.03	** < 0.001**
Neither	14 (8)	32 (16)	40 (24)			
Disagree	4 (2)	23 (11)	15 (9)			
Decreases rates of relapse						
Agree	146 (82)	118 (58)	111 (67)	2	25.04	** < 0.001**
Neither	26 (15)	54 (27)	49 (29)			
Disagree	6 (3)	31 (15)	6 (4)			
Works well in clients with co-occurring mental health disorders						
Agree	143 (79)	103 (52)	99 (60)	2	32.29	** < 0.001**
Neither	32 (18)	71 (36)	64 (38)			
Disagree	5 (3)	25 (12)	3 (2)			
Should be supplemented by mental health counseling						
Agree	155 (90)	172 (84)	129 (77)	2	7.79	**0.020**
Neither	13 (7)	26 (13)	39 (23)			
Disagree	5 (3)	6 (3)	0			
Should be supplemented by participation in peer support groups						
Agree	156 (87)	166 (81)	125 (74)	2	7.80	**0.020**
Neither	18 (10)	33 (16)	42 (25)			
Disagree	5 (3)	5 (3)	2 (1)			
Efficacy is improved by adding mental health counseling						
Agree	166 (91)	175 (88)	132 (79)	2	14.90	**0.001**
Neither	14 (8)	24 (12)	34 (20)			
Disagree	2 (1)	1 (0)	1 (1)			
Appropriate for unstable patients						
Agree	68 (39)	50 (25)	67 (41)	2	19.37	**0.001**
Neither	58 (34)	67 (34)	68 (41)			
Disagree	46 (27)	82 (41)	30 (18)			
Often diverted or misused						
Agree	67 (35)	94 (47)	12 (7)	2	91.95	** < 0.001**
Neither	68 (35)	71 (36)	45 (28)			
Disagree	59 (30)	33 (17)	106 (65)			

^a^P-Values for differences are from Friedman tests

Bold values indicate significance at 0.05

Table 5 depicts the results of the post-hoc Wilcoxon signed-rank tests on the perceived efficacy of BUP, MET, and NTX. Wilcoxon signed-rank tests with Holm-Bonferroni adjustments indicated that providers felt BUP, rather than MET, was more effective in decreasing the risk of death from an opioid overdose (Z = 6.20, *P* < 0.001), decreasing cravings for opioids (Z = 5.08, *P* < 0.001), decreasing rates of relapse (Z = 5.21, *P* < 0.001), working well for clients with co-occurring mental health disorders (Z = 5.93, *P* < 0.001), working well for unstable patients (Z = 3.31, *P* = 0.001), and was less likely to be diverted or misused (Z = − 3.42, *P* = 0.001).

**Table 5:** Post-hoc results from Wilcoxon signed-rank tests on nurse practitioner and physician assistant perceived efficacy of buprenorphine (BUP), methadone (MET), and naltrexone (NTX)

Perceived efficacy	Comparison	Ties	Positive ranks		Negative ranks		Effect size	Z	P^a^
			n	Sum of ranks	n	Sum of ranks			
Decreases risk of death from an opioid overdose	MET vs NTX	88	24	2796	48	6168	− 0.25	− 3.16	**0.002**
	BUP vs NTX	112	28	3634.5	9	1212.5	0.25	3.03	**0.003**
	BUP vs MET	110	56	7987	7	959	0.47	6.20	** < 0.001**
Decreases cravings for opioids	MET vs NTX	103	36	4813	23	3034	0.14	1.73	0.088
	BUP vs NTX	104	40	5052.5	5	662.5	0.42	5.14	** < 0.001**
	BUP vs MET	135	34	5231	3	467	0.39	5.08	** < 0.001**
Decreases rates of relapse	MET vs NTX	98	27	3370.5	36	4819.5	− 0.11	− 1.40	0.156
	BUP vs NTX	103	30	3699	12	1530	0.22	2.68	0.008
	BUP vs MET	107	51	7031.5	10	1386.5	0.40	5.21	** < 0.001**
Works well in clients with co- occurring mental health disorders	MET vs NTX	92	27	3307.5	41	5294.5	− 0.15	− 1.90	0.058
	BUP vs NTX	101	34	4164.5	11	1415.5	0.27	3.30	**0.001**
	BUP vs MET	108	53	7349	7	961	0.46	5.93	** < 0.001**
Should be supplemented by mental health counseling	MET vs NTX	135	19	2831	8	1192	0.17	2.12	0.052
	BUP vs NTX	116	17	2167.5	7	916.5	0.17	1.99	0.064
	BUP vs MET	144	13	1989	6	937	0.12	1.57	0.167
Should be supplemented by participation in peer support groups	MET vs NTX	132	20	2955	10	1470	0.14	1.84	0.080
	BUP vs NTX	120	19	2530.5	7	940.5	0.19	2.34	0.028
	BUP vs MET	149	13	2067	7	1123	0.10	1.32	0.263
Efficacy is improved by adding mental health counseling	MET vs NTX	135	19	2805	5	735	0.23	2.86	0.006
	BUP vs NTX	125	19	2584	4	567	0.25	3.07	**0.003**
	BUP vs MET	150	13	2077	7	1133	0.10	1.32	0.259
Appropriate for unstable patients	MET vs NTX	72	26	3069	63	7344	− 0.30	− 3.81	** < 0.001**
	BUP vs NTX	69	32	3382	41	4356	− 0.09	− 1.06	0.300
	BUP vs MET	97	46	6071	20	2542	0.26	3.31	**0.001**
Often diverted or misused	MET vs NTX	55	95	10,136	7	727	0.68	8.51	** < 0.001**
	BUP vs NTX	61	78	8677.5	17	1677.5	0.52	6.44	** < 0.001**
	BUP vs MET	82	34	4328	66	8922	− 0.25	− 3.42	**0.001**

^a^Holm-Bonferroni method adjusted p-values for 27 comparisons

Bold values indicate a test is significant once the corrected p-value is less than the significance level of 0.05

BUP was perceived to be more effective than NTX in decreasing the risk of death from an opioid overdose (Z = 3.03, *P* = 0.003), decreasing cravings for opioids (Z = 5.14, *P* < 0.001), and working well in individuals with co-occurring mental health disorders (Z = 3.30, *P* = 0.001). Providers also perceived the efficacy of BUP to improve when supplemented with counseling (Z = 3.07, *P* = 0.003), and felt that it is was more likely to be diverted or misused (Z = 6.44, *P* < 0.001) when compared to NTX. Lastly, when comparing to MET, providers were more likely to agree that NTX decreases the risk of death from an opioid overdose (Z = − 3.16, *P* = 0.002), is appropriate for unstable patients (Z = − 3.81, *P* < 0.001), and is less likely to be diverted or misused.

## Discussion

Both waivered and non-waivered respondents in our sample overwhelmingly agreed BUP decreases risk of overdose, relapse, and cravings and BUP should be supplemented with counseling and peer support groups. Nevertheless, waivered respondents were more likely to believe BUP is efficacious, potentially due to the waiver education process or direct experience with BUP prescribing. Alternatively, practitioners with positive beliefs about BUP efficacy may be more likely to pursue a waiver.

As in a recent study of physician-reported barriers [18], our results suggest perceptions of barriers differ by waiver status – an important finding indicating that different policy approaches are needed to address BUP expansion for non-waivered versus waivered NPs/PAs. Waivered respondents indicated significantly fewer prescribing barriers than did non-waivered respondents, suggesting that perceptions of barriers may prevent some from seeking a waiver. Since federal guidelines released in 2021 will squarely address training barriers related to obtaining a waiver [19], it will be important to observe how the lack of training requirements will impact willingness to obtain a waiver. The lack of a training requirement for prescribing to fewer than 30 patients may increase prescribing behavior among NPs and PAs who wish to “dabble” in BUP without making BUP a key part of their practice [20]. It is also possible that since waivered respondents in our sample were more likely to work in specialty SUD settings, they may face fewer institutional barriers.

Problematically, non-waivered respondents had concerns that BUP would unfavorably affect patient mix, resembling a finding in a study of physician BUP barriers [21], suggesting stigmatization of OUD patients across multiple practitioner types. A qualitative study of physicians found that patient mix concerns were driven by fears that OUD patients would cause non-OUD patients to leave the practice—an indication that practitioners believe patients also stigmatize each other [22]. Significantly more research is needed regarding evidence-based interventions to decrease stigma toward OUD patients.

Interestingly, even though BUP only requires partial detoxification, a large minority of non-waivered respondents felt insufficient access to detoxification was a primary barrier—a novel finding regarding BUP barriers. It is possible that non-waivered practitioners are unaware that BUP does not require complete detoxification. Surprisingly, even though a previous study of waivered NPs/PAs from 2020 found diversion/misuse concerns were a primary barrier [14], as did a study of non-waivered physicians [21], that barrier was not prominent in our results.

We also compared perceptions of MOUD efficacy across medications without disaggregating based on waiver status (due to sample size limitations.) Respondents were more likely to believe that NTX reduces overdose deaths as compared to MET. MET has a stronger evidence base than does NTX, particularly with respect to preventing overdose deaths in real-world (i.e., non-clinical trial) settings [1]. Consistent with person-centered care principles, NPs/PAs should offer all options to patients, either by directly prescribing the MOUD or referring patients to MOUD. It appears, however, that NPs/PAs may require additional education about the relative efficacy of different MOUD options, particularly MET. It is possible that most NPs/PAs have less experience with MET prescribing/administration, since MET is not available in office-based settings. Lack of familiarity with MET could contribute to misperceptions about MET efficacy.

Our study is unique in that it examined perceptions of MOUD efficacy for unstable patients. We did not provide a definition for “unstable patients” in our study, so future work should examine how clinicians define “unstable,” including specific patient characteristics clinicians associate with stability. Our study also examined perceptions of efficacy of MOUD for patients with co-occurring disorders, finding significant differences by type of medication. Patients with OUD often have co-occurring mental health disorders and/or other substance use disorders (e.g., methamphetamine use disorder) [23–26], and perceptions of MOUD efficacy for patients with co-occurring disorders could impact MOUD prescribing behavior. Future studies should examine MOUD prescribing behavior to patients with co-occurring disorders and the relationship of such prescribing behavior with NP/PA training in dual diagnosis treatment.

Lastly, our study found that respondents overwhelmingly believed counseling and peer support improved efficacy of all MOUDs examined. We also found that a large minority of providers felt that insufficient behavioral support services were a barrier to MOUD prescribing, suggesting that some respondents feel they cannot or should not prescribe MOUD if patients are not obtaining adequate behavioral support services. With respect to BUP, the literature is mixed regarding effectiveness of adding counseling to MOUD services [27, 28], and we are not aware of studies comparing efficacy of NTX with counseling to NTX without counseling. The American Society of Addiction Medicine’s OUD treatment practice guidelines recommend that clinicians offer behavioral health services alongside MOUD but urge against requiring behavioral health services [29]. Therefore, we recommend that policymakers and healthcare administrators urge NPs/PAs to offer behavioral support but not require it for MOUD treatment.

The study has several limitations, including a non-representative sample, low response rate, and small sample size. Although the survey was piloted, it was not validated. NPs in the sample were more likely to be waivered than PAs, potentially biasing the opinion of waivered providers toward that of NPs. Additionally, nonparametric tests resulted in lost statistical power. Our ability to draw inferences is limited by small effect sizes. Importantly, we did not examine the role of scope of practice laws as a barrier to BUP prescribing, even though previous research has found scope of practice laws impact waiver uptake [30] and prescribing behavior among NPs/PAs [31].

## Conclusion

NPs/PAs have significantly influenced expansion of BUP availability in the U.S., particularly in rural areas. Nevertheless, they face barriers to prescribing BUP. Future qualitative research should explore how NPs/PAs feel barriers identified in this study could be addressed. Since NPs/PAs in some states are subject to scope of practice laws requiring collaboration with or supervision by a physician, approaches to addressing barriers for NPs/PAs may differ not only by waiver status but also by state policies. Higher perceptions of NTX efficacy as compared to MET efficacy is problematic, as the evidence base suggests MET is more effective at preventing overdose [1]. Therefore, while NPs/PAs should have knowledge about all MOUD options, additional information should be provided to NPs/PAs about relative efficacy of treatments (e.g., during continuing medical education seminars) regardless of their waiver status—particularly since NPs/PAs can refer patients to other MOUD providers even if they are not prescribing MOUD themselves.

## Data Availability

Deidentified data is available upon request by contacting the lead author at barbara.andraka@ucf.edu.

## Abbreviations

BUP: Buprenorphine treatment
DATA waiver: Federal waiver required for practitioners to prescribe buprenorphine in office-based settings
MOUD: Medication for opioid use disorder
MET: Methadone treatment
NP: Nurse practitioner
NTX: Naltrexone treatment
PA: Physician assistant
